# Grouping-Based and Position-Based Phase Optimization for RIS-Assisted Millimeter-Wave Vehicular Communications

**DOI:** 10.3390/s26154862

**Published:** 2026-08-02

**Authors:** Zongliang Xu, Guicai Yu, Yingcong Luo

**Affiliations:** 1 School of Intelligent Science and Engineering, Qinghai Minzu University, Xining 810007, China; 2023087@qhmu.edu.cn (Z.X.); 15972637020@163.com (Y.L.); 2 National Demonstration Center for Experimental Teaching of Communication Engineering, Qinghai Minzu University, Xining 810007, China

**Keywords:** reconfigurable intelligent surface, millimeter-wave vehicular communications, low-overhead phase optimization, channel reconstruction, successive refinement algorithm, position-aided optimization

## Abstract

Millimeter-wave vehicular communication links are prone to blockage and suffer from severe path loss, and high mobility leads to rapidly time-varying channels. In addition, large-scale reconfigurable intelligent surface (RIS) arrays impose substantial channel-estimation overhead and phase-optimization complexity. To address these issues, a group-based and position-aided phase-optimization method is proposed for RIS-assisted millimeter-wave vehicular communications. First, an RIS-assisted uplink system is modeled with a multi-antenna base station (BS), an RIS configured as a uniform planar array (UPA) and a single-antenna vehicular terminal. Channel expressions are formulated for the direct vehicle–BS link, the vehicle–RIS link and the RIS–BS link. Rician fading, line-of-sight (LoS)-dominated millimeter-wave propagation, mobility-induced Doppler shifts and a standardized path-loss model for urban microcell street-canyon scenarios are incorporated to characterize the RIS-assisted vehicular cascaded channel. Based on this model, an optimization problem for the RIS phase-shift matrix is formulated under discrete phase-shift constraints to maximize the achievable rate per unit bandwidth. To avoid the exponential increase in complexity caused by conventional exhaustive search as the number of RIS reflecting elements increases, a successive refinement algorithm is introduced to derive an equivalent channel-gain expression. The original phase-optimization problem is then transformed into an element-wise iterative update process, thereby reducing the computational complexity of large-scale RIS phase configuration. To further reduce the reliance on full channel state information (CSI), two low-overhead phase-optimization schemes are designed. In the group-based scheme, the RIS reflecting elements are partitioned into several subgroups, with all elements in each subgroup constrained to share the same phase shift. This design reduces both the channel-estimation dimensionality and the number of optimization variables. In the position-aided scheme, the spatial coordinates of the BS, RIS and vehicle are used to derive the link distances and the associated angles of arrival and departure. Based on these geometric parameters, the vehicle–RIS–BS cascaded channel is reconstructed and a corresponding phase-alignment strategy is designed. The simulation results demonstrate that both proposed schemes achieve rates of approximately 6.5 bits $s^{-1}{Hz}^{-1}$ at a transmit power of 30 dBm and outperform existing phase-optimization techniques. When the successive refinement algorithm is applied, the computation time required for phase optimization with a 256-element RIS remains below 0.01 s. Under high-mobility conditions, both proposed schemes approach the performance upper bound achieved with perfect CSI, demonstrating strong robustness to channel variations.

## 1. Introduction

With the rapid development of intelligent transportation systems (ITSs), the demand for high-reliability and high-data-rate communication in high-mobility vehicular scenarios has become increasingly prominent, motivating extensive research on vehicular communication technologies in both academia and industry [1]. Vehicular communications generally include vehicle-to-vehicle (V2V), vehicle-to-infrastructure (V2I), vehicle-to-pedestrian (V2P) and vehicle-to-network (V2N) communications. These communication modes are collectively referred to as vehicle-to-everything (V2X) communications. However, existing fifth-generation (5G) mobile networks still fall short of fully satisfying the rapidly growing performance requirements of V2X communications [2], particularly in dense vehicular networking, autonomous driving and intelligent transportation applications [3]. Moreover, rapidly time-varying wireless channels arising from high mobility remain a major challenge to achieving high-capacity, ultra-reliable and low-latency V2X communications [4]. Two major technical challenges also remain in 5G networks. First, massive multiple-input multiple-output (massive MIMO) systems require numerous active antenna elements and radio-frequency (RF) chains, resulting in high hardware costs and energy consumption [5]. The second challenge is associated with millimeter-wave propagation. Millimeter-wave signals operate in high-frequency bands and have short wavelengths, leading to severe path loss and penetration loss [6]. These propagation limitations make it difficult for existing 5G networks to support the development of green and sustainable cellular networks.

Reconfigurable intelligent surface (RIS) technology is regarded as a key enabling technology for sixth-generation (6G) mobile communications owing to its ability to intelligently reconfigure the wireless propagation environment at low hardware cost and with low energy consumption [7]. Accordingly, RIS-assisted vehicular communication systems have attracted considerable research attention in recent years as a promising solution to support the development of sustainable cellular networks and meet the stringent requirements of vehicular communications [8]. An RIS is composed of a large number of low-cost passive elements [9]. Unlike conventional phased-array antennas, an RIS does not actively transmit electromagnetic waves [10]. Instead, each element can independently adjust the phase shift in the incident electromagnetic wave. By adaptively tuning the RIS phase shifts, the robustness of the communication system can be improved, and this process is commonly referred to as passive beamforming [11]. Unlike conventional transmit and receive antennas, an RIS can flexibly reconfigure the wireless propagation channel by creating controllable reflected paths to bypass obstacles, thereby improving the received signal quality. Unlike conventional amplify-and-forward (AF) relays, an RIS passively reflects incident signals and reconfigures its reflection coefficients in real time while consuming little energy and introducing negligible noise amplification [12]. An RIS with a large number of reflecting elements can provide substantial array gain and passive beamforming gain without incurring excessive hardware costs.

In recent years, various optimization frameworks and algorithms have been proposed to address the phase-optimization problem in RIS-assisted vehicular communications, with the aim of improving system capacity, energy efficiency and robustness. An RIS-UAV-assisted vehicular communication network was proposed in [13], in which the RIS phase shifts and UAV trajectory were jointly optimized to maximize the achievable rate. An alternating optimization algorithm was adopted to address the resulting nonconvex problem. However, this method relies on perfect channel state information (CSI) and involves high computational complexity in high-mobility scenarios, thereby limiting its real-time adaptability to dynamic channel variations. For an RIS-assisted full-duplex 6G-V2X communication network, the phase-shift matrices of two RISs were jointly optimized in [14] to maximize the achievable sum rate. A deep reinforcement learning algorithm based on proximal policy optimization (PPO) was adopted to address the resulting nonconvex continuous-action-space optimization problem. Although this method reduces online optimization complexity, it still incurs considerable training overhead and depends strongly on accurate CSI in high-mobility scenarios. Consequently, its real-time performance and robustness require further improvement for practical deployment. A dynamic multi-RIS-assisted vehicle-to-infrastructure (V2I) network was investigated in [15]. A method based on deep learning (DL) was used to optimize the phase-shift configuration to maximize energy efficiency and improve transmission performance. The algorithm approximated the complex optimization problem using neural networks and achieved fast convergence. However, it requires extensive training data and exhibits limited generalization capability under channel uncertainties arising from blockage and interference. An element-selection-based phase-optimization method was introduced in [16] for an RIS-assisted multi-vehicle network. In this method, a subset of RIS elements was selected for phase-shift adjustment to maximize the sum rate and reduce the number of optimization variables. However, this method does not sufficiently account for severe path loss and line-of-sight (LoS)-dominated propagation characteristics in millimeter-wave bands. This limitation may lead to performance degradation over long-distance vehicular links. A low-latency V2I system assisted by a simultaneously transmitting and reflecting reconfigurable intelligent surface (STAR-RIS) was investigated in [17]. In this system, the phase shifts and power allocation were jointly optimized to minimize the maximum delay among vehicles. A differential evolution (DE) algorithm was adopted to address the resulting optimization problem. Although this framework considers the simultaneous transmission and reflection modes of STAR-RIS, it still incurs high computational overhead and relies on the assumption of a static environment. Therefore, the complexities introduced by vehicle clustering and multi-hop transmission remain insufficiently addressed. An energy-efficient RIS-assisted multi-cell non-orthogonal multiple-access (NOMA) system was proposed in [18]. In this system, the transmit beamforming vectors and RIS phase shifts were jointly optimized to maximize the system energy efficiency. A block coordinate descent (BCD) algorithm was adopted to decompose the resulting optimization problem into tractable subproblems.

In terms of channel estimation and beamforming, an MMSE-interpolation-based channel-estimation technique was proposed in [19] to address the irreducible error floor caused by the conventional block-fading assumption in RIS channel estimation under high Doppler shifts. In [20], a location-information-assisted compressive-sensing channel-estimation algorithm was proposed to reduce the complexity of channel estimation. In this method, readily available device-location information in the Internet of Vehicles was used to construct the system model and derive the optimal RIS phase-shift matrix, thereby reducing the channel-training overhead and estimation complexity. In [21], a low-complexity passive beamforming scheme was developed by combining the symmetric deployment of roadside RISs with offline/online training. In [22], an IRS-assisted joint beamforming design was proposed, in which the base-station transmit precoding matrix and the IRS reflection phase-shift matrix were jointly optimized to maximize the spectral efficiency of V2I users.

In terms of security and positioning, the physical-layer security performance of a dual-RIS-assisted V2V NOMA system was investigated in [23], where analytical expressions for relevant security metrics were derived based on a specific fading model, and the effectiveness of the system in improving the security and reliability of intelligent transportation systems was verified. In [24], an RIS-assisted secure transmission model was established for the coexistence scenario of V2I and V2V communications in the presence of eavesdroppers. In [25], an RIS-enhanced millimeter-wave positioning and communication scheme was proposed to improve positioning accuracy.

Although these studies have advanced RIS phase optimization, several common limitations remain. These limitations can be summarized as follows. (1) The optimization algorithms adopted in these studies typically incur high computational complexity, particularly for large-scale RIS arrays. (2) Many studies assume perfect CSI and therefore overlook channel-estimation overhead and mobility-induced Doppler spread in high-mobility vehicular scenarios. (3) Position information and grouping strategies are rarely exploited, resulting in insufficient robustness in the presence of dynamic blockage and over long-distance vehicular links. To address these limitations, this study proposes a group-based and position-aided phase-optimization method for RIS-assisted millimeter-wave vehicular communications. In this method, a successive refinement algorithm is employed to reduce the computational complexity of phase configuration for large-scale RIS arrays, thereby enabling low-overhead and high-performance RIS-assisted millimeter-wave vehicular communications.

The main contributions of this paper are summarized as follows:

(1) An RIS-assisted millimeter-wave vehicular uplink system model is established. The system comprises a multi-antenna base station (BS), an RIS configured as a uniform planar array (UPA) and a single-antenna vehicular terminal. Channel models are formulated for the direct vehicle–BS link, the vehicle–RIS link and the RIS–BS link. The model incorporates LoS-dominated millimeter-wave propagation, mobility-induced Doppler shifts and distance-dependent path loss, thereby providing a realistic characterization of high-mobility vehicular channels.
(2) A discrete phase-optimization problem is formulated for the RIS reflection matrix by taking the achievable rate per unit bandwidth as the optimization objective. This formulation clarifies how the RIS phase-shift matrix affects the vehicle–BS transmission rate. To avoid the high computational burden caused by exhaustive search over discrete phase-shift states, a successive refinement algorithm is introduced. The channel-gain expression is reformulated into an equivalent element-wise iterative update form, thereby reducing the computational complexity of phase configuration for large-scale RIS arrays.
(3) Two low-overhead RIS phase-optimization schemes are designed to reduce the system’s reliance on full CSI. In the group-based scheme, the RIS reflecting elements are partitioned into multiple subgroups, and all elements within each subgroup share the same phase shift. This design reduces both the channel-estimation dimensionality and the number of optimization variables. In the position-aided scheme, the spatial geometric parameters of the BS, RIS and vehicle are used to derive the link distances and angular information required for cascaded channel reconstruction. The RIS phase shifts are then optimized to achieve coherent combining of the reflected signals at the BS.
(4) Simulations were conducted under various transmit powers, RIS array sizes, vehicle speeds, and phase quantization resolutions. The results demonstrate that the two proposed optimization schemes substantially reduce the channel-estimation overhead while maintaining a high achievable rate. Compared with conventional algorithms, the proposed schemes achieve higher achievable rates, particularly in large-scale RIS-assisted scenarios. The successive refinement algorithm also exhibits rapid convergence and requires less than 0.01 s for each computation when a 256-element RIS is considered. These results indicate that the proposed schemes are suitable for low-overhead and high-performance millimeter-wave vehicular communications.

## 2. System Model

This paper considers an uplink RIS-assisted vehicular communication system comprising a BS, an RIS, and a single-antenna vehicular terminal. The BS is equipped with a uniform linear array (ULA), whereas the RIS consists of multiple passive reflecting elements arranged in a uniform linear structure. In practical deployment scenarios, the BS, RIS, and vehicular terminal are usually installed at different heights. Specifically, the BS is typically located at the highest position, followed by the RIS, while the vehicular terminal is located at a relatively low height. Accordingly, the involved channels are assumed to be dominated by LoS propagation and are modeled as Rician fading channels. To characterize the path loss, the 3GPP TR 38.901 UMi street-canyon path-loss model is adopted [26]. The path-loss expressions for LoS and non-line-of-sight (NLoS) conditions are given as follows:

(1) $$PL_{\mathrm{LOS}}=32.4+21\log_{10}(d)+20\log_{10}(f_{c})$$

(2) $$PL_{\mathrm{NLOS}}=35.3\log_{10}\left(d\right)+22.4+21.3\log_{10}\left(f_{c}\right)-0.3(h_{\mathrm{UT}}-1.5)$$

where d is the distance between the transmitter and receiver, $f_{c}$ is the carrier frequency, and $h_{\mathrm{UT}}$ is the height of the user terminal.

**Figure 1:**
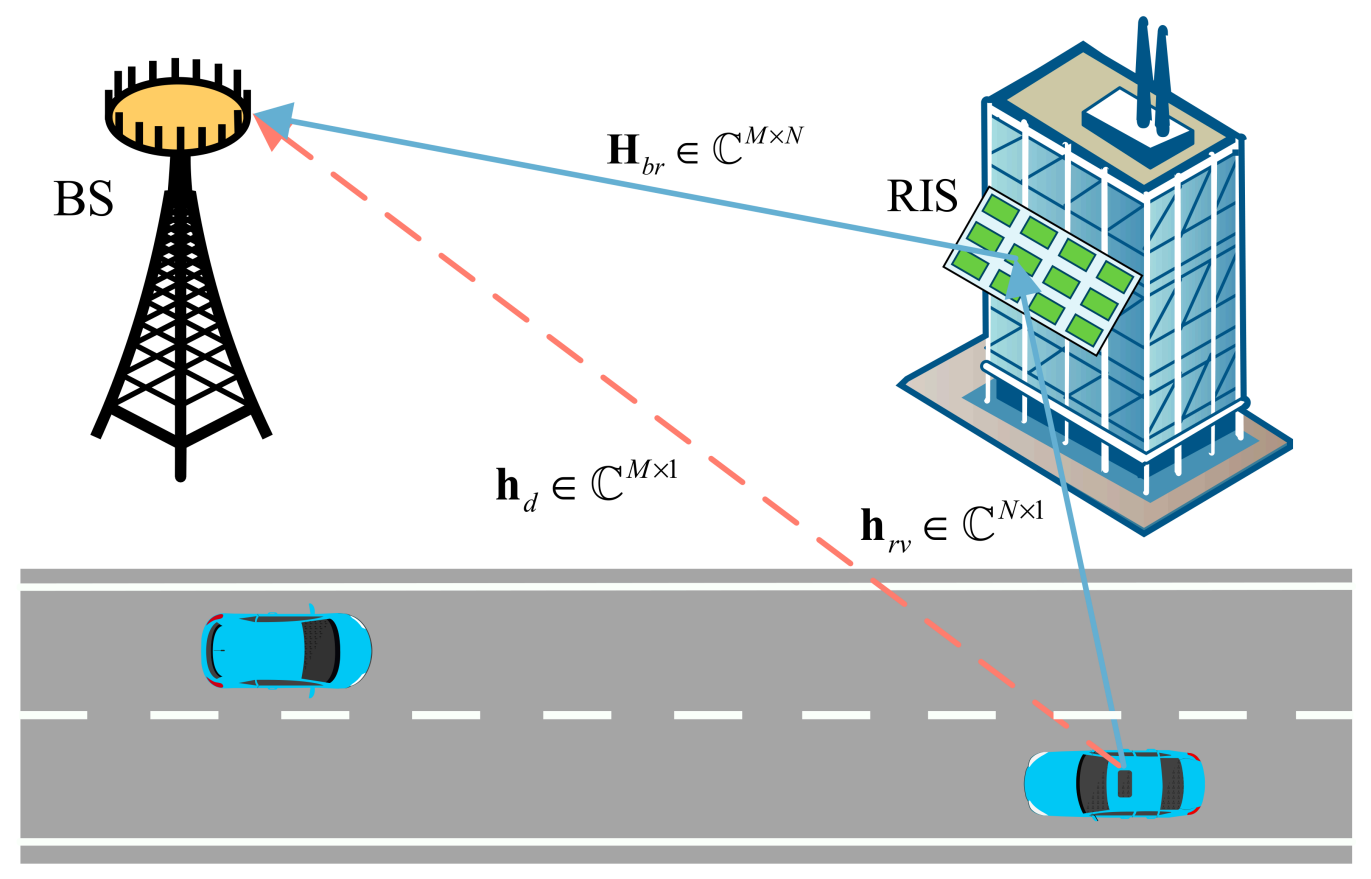
RIS-assisted in-vehicle communication diagram.

The RIS is equipped with a uniform planar array featuring $N=N_{y}\times N_{z}$ reflectors to assist in communication (see Figure 1).

In the uplink, the direct vehicle-to-BS channel is modeled as $h_{d}\in {\mathbb{C}}^{M\times 1}$. The RIS-assisted link consists of cascaded propagation channels; accordingly, the MS-to-RIS channel is modeled as $h_{rv}\in {\mathbb{C}}^{N\times 1}$ and the RIS-to-BS channel as $H_{br}\in {\mathbb{C}}^{M\times N}$. In the mathematical formulation the vehicular terminal is treated as the mobile station (MS). The subscript MS is therefore used for all quantities associated with the vehicular terminal. The direct MS-to-BS channel can then be expressed as follows:

(3) $$h_{d}(t)=\sqrt{\beta_{d}}\left[\sqrt{\frac{K_{d}}{K_{d}+1}}{\bar{h}}_{d,\mathrm{LoS}}(t)+\sqrt{\frac{1}{K_{d}+1}}{\tilde{h}}_{d,\mathrm{NLoS}}(t)\right]$$

where $K_{d}$ is the Rician factor, $\beta_{d}$ is the linear large-scale channel gain, ${\bar{h}}_{d,\mathrm{LoS}}(t)$ is the deterministic LoS component, and ${\tilde{h}}_{d,\mathrm{NLoS}}(t)\sim CN(0,I_{M})$ is the stochastic NLoS component. The pure-LoS model is recovered only in the limiting case $K_{d}\to \infty$.

The deterministic LoS component in Equation (3) incorporates the mobility-induced Doppler phase and the BS array response and is written as

(4) $${\bar{h}}_{d,\mathrm{LOS}}(t)=e^{j2\pi f_{d,{\bar{h}}_{d}}t}\alpha_{\mathrm{BS}}(\theta )\alpha_{\mathrm{MS}}^{H}(\varphi )$$

where $f_{d,{\bar{h}}_{d}}=v\cos(\beta_{{\bar{h}}_{d}})/\lambda$ denotes the Doppler frequency shift, v denotes the moving speed of the vehicular terminal, $\beta_{{\bar{h}}_{d}}$ denotes the angle between the vehicle-motion direction and the signal-propagation direction, θ denotes the angle of departure (AoD) at the BS, and φ denotes the angle of arrival (AoA) at the vehicular terminal.

Considering that the BS is equipped with M antennas and the vehicular terminal is equipped with a single antenna, the BS array response vector $\alpha_{\mathrm{BS}}(\theta )$ and the antenna response at the vehicular terminal can be expressed as follows [27]:

(5) $$\alpha_{\mathrm{BS}}(\theta ,M)={\left[1,e^{j\frac{2\pi }{\lambda }d\sin(\theta )},\ldots ,e^{j\frac{2\pi }{\lambda }(M-1)d\sin(\theta )}\right]}^{T}$$

(6) $$\alpha_{\mathrm{MS}}(\varphi ,1)={\left[1,e^{j\frac{2\pi }{\lambda }d\sin(\varphi )}\right]}^{T}$$

where λ denotes the signal wavelength, d=λ/2 denotes the antenna spacing, θ denotes the incident angle at the BS, and φ denotes the AoA at the MS.

Since the RIS is modeled as a uniform planar array, a UPA, its array response vector can be expressed as the Kronecker product of two one-dimensional array response vectors along the z- and y-axes. The array responses along the z- and y-axes are then obtained from the normalized one-dimensional array response vector defined in Equation (5) as follows:

(7) $$\alpha_{1}\left(\theta ,M_{z}\right)={\left[1,e^{j\pi \sin(\theta )},\ldots ,e^{j\pi (M_{z}-1)\sin(\theta )}\right]}^{T}$$

(8) $$\alpha_{2}\left(\phi ,M_{y}\right)={\left[1,e^{j\pi \sin(\phi )\cos(\theta )},\ldots ,e^{j\pi (M_{y}-1)\sin(\phi )\cos(\theta )}\right]}^{T}$$

The RIS array response vector can then be constructed from the one-dimensional array response vectors in Equations (7) and (8) as follows:

(9) $$\begin{array}{l} \alpha_{\mathrm{RIS}}\left(\theta ,\phi \right)=\alpha_{1}\left(\theta ,M_{z}\right)⊗\alpha_{2}\left(\phi ,M_{y}\right)\\ \;\;\;\;\;\;\;\;\;\;\;\;\;\;\;\;={\left\{1,e^{j\pi [\sin(\theta )+\sin(\phi )\cos(\theta )]},\ldots ,e^{j\pi [(M_{z}-1)\sin(\theta )+(M_{y}-1)\sin(\phi )\cos(\theta )]}\right\}}^{T} \end{array}$$

where θ∈[0, π] and ϕ∈[0, 2π] are the elevation and azimuthal angles of the RIS, respectively.

**Figure 2:**
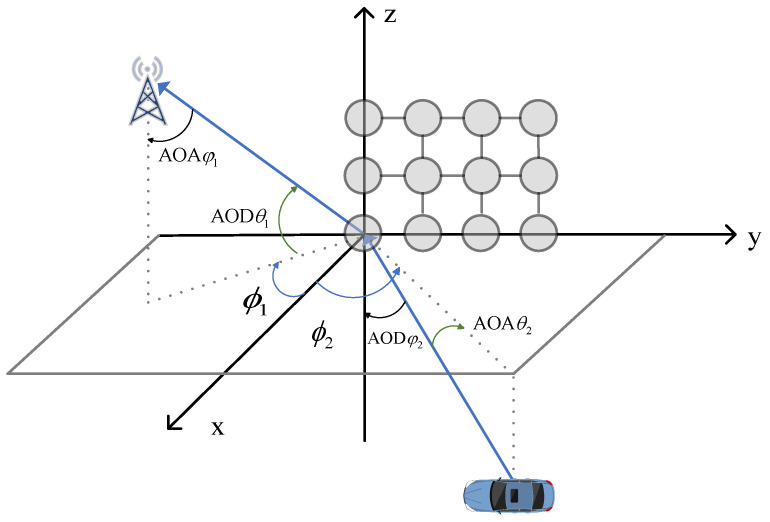
Three-dimensional RIS-assisted in-vehicle communication.

To derive the explicit expression for the cascaded channel model, a three-dimensional Cartesian coordinate system is established with the center of the RIS located at the origin as shown in Figure 2.

Based on the array response definitions, the complete RIS-to-BS channel $H_{br}$ and vehicle-to-RIS channel $h_{rv}$, including both LoS and NLoS components, are expressed as [28]

(10) $$H_{br}(t)=\sqrt{\beta_{br}}\left[\sqrt{\frac{K_{br}}{K_{br}+1}}{\bar{H}}_{br,\mathrm{LoS}}(t)+\sqrt{\frac{1}{K_{br}+1}}{\tilde{H}}_{br,\mathrm{NLoS}}(t)\right]$$

(11) $$h_{rv}(t)=\sqrt{\beta_{rv}}\left[\sqrt{\frac{K_{rv}}{K_{rv}+1}}{\bar{h}}_{rv,\mathrm{LoS}}(t)+\sqrt{\frac{1}{K_{rv}+1}}{\tilde{h}}_{rv,\mathrm{NLoS}}(t)\right]$$

where $\beta_{br}$ and $\beta_{rv}$ are the linear large-scale gains and $K_{br}$ and $K_{rv}$ are the corresponding Rician factors. The deterministic terms ${\bar{H}}_{br,\mathrm{LoS}}(t)$ and ${\bar{h}}_{rv,\mathrm{LoS}}(t)$ are constructed from the link-specific AoD/AoA array responses and Doppler phases. The stochastic components satisfy $\mathrm{vec}({\tilde{H}}_{br,\mathrm{NLoS}})\sim CN(0,I_{MN})$ and ${\tilde{h}}_{rv,\mathrm{NLoS}}\sim CN\;\left(0,I_{N}\right)$. Consequently, Equations (3), (10), and (11) retain the scattered NLoS energy rather than treating a finite-K Rician channel as a pure-LoS channel.

(12) $${\bar{H}}_{br,\mathrm{LoS}}(t)=e^{j2\pi f_{D,{\bar{H}}_{br,\mathrm{LoS}}}t}\alpha_{\mathrm{BS}}\left(\varphi_{1}^{\mathrm{BS}},M\right)\alpha_{\mathrm{RIS}}^{H}\left(\theta_{1}^{\mathrm{RIS}},\phi_{1}^{\mathrm{RIS}}\right)$$

(13) $${\bar{h}}_{rv,\mathrm{LoS}}(t)=e^{j2\pi f_{D,rv}t}a_{\mathrm{RIS}}\left(\theta_{rv},\phi_{rv}\right)$$

where in Equation (12), $\theta_{1}^{\mathrm{RIS}}$ and $\phi_{1}^{\mathrm{RIS}}$ represent the elevation angle and azimuth angle from RIS to BS respectively; the whole is the departure angle of the RIS end, and $\varphi_{1}^{\mathrm{BS}}$ represents the arrival angle of the BS end. In Equation (13), $\theta_{2}^{\mathrm{RIS}}$ and $\phi_{2}^{\mathrm{RIS}}$ represent the elevation angle and azimuth angle from the vehicle to the RIS, respectively; the whole is the arrival angle of the RIS end, and $\varphi_{2}^{\mathrm{MS}}$ represents the departure angle of the vehicle end.

## 3. Design of Algorithm for Phase Optimization

An ideal RIS reflection model is adopted in this study, in which hardware impairments such as nonlinear distortion and additive noise are neglected. The signal reflected by the n-th RIS element is denoted by ${\hat{y}}_{n}$ and modeled as the product of the corresponding incident signal $x_{n}$ and the complex reflection coefficient $\beta_{n}$. This relationship can be expressed as follows [29]:

(14) $${\hat{y}}_{n}=\beta_{n}e^{j\theta_{n}}{\hat{x}}_{n},n\in N$$

where $\beta_{n}\in [0,1]$ and $\theta_{n}\in [0,2\pi )$ denote the reflection amplitude coefficient and phase-shift angle of the n-th RIS element, respectively. An RIS with *N* elements can therefore be represented by an N×N diagonal reflection matrix Θ according to the element-wise reflection relationship in Equation (14). The reflected signal vector is given by $\hat{y}=\Theta \hat{x}$. where $\Theta =\mathrm{diag}(\beta_{1}e^{j\theta_{1}},\ldots ,\beta_{N}e^{j\theta_{N}})$, $\hat{x}={\left[{\hat{x}}_{1},\ldots ,{\hat{x}}_{N}\right]}^{T}$, $\hat{y}={\left[{\hat{y}}_{1},\ldots ,{\hat{y}}_{N}\right]}^{T}$.

Ideally, each RIS element can independently control both the reflection amplitude and phase, thereby enabling flexible channel reconfiguration and supporting channel estimation, energy harvesting, and system optimization. However, owing to hardware cost and implementation complexity, practical RIS deployments often adopt a fixed-amplitude reflection design and retain only phase control. This simplified design substantially reduces hardware cost while preserving the dominant performance gain provided by RIS phase adjustment. To fully exploit the RIS performance, the reflection amplitude coefficient is set to unity, i.e., β=1, to enhance the effective cascaded channel gain. The reflecting elements of the RIS impose phase shifts on the incident signal. In this section, the phase shift in each RIS element is constrained to one of L discrete values obtained by uniformly quantizing the interval $\left[0,2\pi \right]$. Accordingly, the discrete phase-shift set for each RIS reflecting element is given by

(15) F={0,Δθ,…,(L−1)Δθ}

where Δθ=2π/L.

Let $\theta_{i}\in F$ denote the phase shift in the i-th RIS reflecting element. When the signal transmitted by the vehicular terminal is x, the signal received at the BS is given by

(16) $$y=(h_{d}+H_{br}\Theta h_{rv})x+n$$

where $n=[n_{1},n_{2},\ldots ,n_{K}]$, and $n_{k}\sim CN(0,N_{0})$ is the additional Gaussian white noise on the BS antenna, which has a mean of zero and a variance of $N_{0}$. The linear beamforming vector w is used at the BS side to decode x, which can be expressed as

(17) $$\hat{y}=w^{H}(h_{d}+H_{br}\Theta h_{rv})x+w^{H}n$$

To decode the transmitted signal x, the effective combined channel gain is defined as $w=h_{d}+H_{br}\Theta h_{rv}$. When the transmit power is P, the received signal-to-noise ratio (SNR) is given by

(18) $$\mathrm{SNR}=\frac{P{\left\|h_{d}+H_{br}\Theta h_{rv}\right\|}^{2}}{N_{0}}$$

The bandwidth-normalized channel capacity represents the maximum amount of information that can be transmitted per unit bandwidth. Using the SNR expression in Equation (18), this achievable rate can be expressed as

(19) $$R=\log_{2}\left(1+\frac{P{\left\|h_{d}+H_{br}\Theta h_{rv}\right\|}^{2}}{N_{0}}\right)\mathrm{bit}\;s^{-1}\;{\mathrm{Hz}}^{-1}.$$

Equation (19) indicates that the achievable rate depends on the RIS reflection matrix Θ. Therefore, the achievable rate can be improved by properly optimizing Θ. This process is also referred to as passive beamforming or phase optimization. In this study, the RIS phase-optimization problem is formulated as a rate-maximization problem by selecting the optimal discrete phase shifts.

(20) $$\begin{array}{l} \mathrm{maximize}\;R\\ \mathrm{subject}\;\mathrm{to}\;\theta_{i}\in F,\;\mathrm{for}\;i\;=\;1,2,\ldots ,N. \end{array}$$

Due to the discrete nature of the phase shifts, the optimization problem in Equation (20) is nonconvex. Although an exhaustive search can be used to maximize the achievable rate, it requires examining $N^{L}$ candidate phase-shift configurations. Such a search becomes computationally prohibitive for large RIS arrays. To reduce the computational complexity, an expanded channel-gain expression is first derived. The phase shifts are then optimized using the successive refinement algorithm in [30] (see Algorithm 1). We define $\Phi =H_{br}\mathrm{diag}(h_{rv})$, $v={\left[\exp(j\theta_{1}),\exp(j\theta_{2}),\ldots ,\exp(j\theta_{N})\right]}^{T}$, $A=\Phi^{H}\Phi$, and $b=\Phi^{H}h_{d}$. The channel-gain expression then simplifies to

(21) hd+HbrΘhrv2=vHAv+2Re{vHb}+hd2

A detailed proof of Equation (21) is provided in Appendix A. For a fixed RIS geometry, the overall channel gain can be decomposed into the contributions of individual reflecting elements. The channel-gain contribution of the n-th reflecting element is given by

(22) 2Re{vn*kn}+τn

where $k_{n}=\sum_{j\neq n}A_{nj}v_{j}+b_{n}$, τn=∑j≠n∑i≠nvi*Aijvj+2Re{∑i≠nvi*bi}+Ann+hd2, where $k_{n}$ and $\tau_{n}$ are constants, $A_{ij}$ denotes the (i,j)-th element of A, and $b_{i}$ denotes the i-th element of b.

The computational complexity of Algorithm 1 primarily depends on the number of RIS reflecting elements N, the number of discrete phase levels L, the number of BS antennas M, and the number of outer iterations K. In the inner loop, each RIS element is optimized by computing $k_{n}$ and selecting the nearest discrete phase. The corresponding time complexity is $O(N^{2}+NL)$. In each outer iteration, computing the achievable rate requires an additional complexity of O(MN). Therefore, the overall computational complexity is $O(N^{2}+NL+MN)$. This polynomial complexity is significantly lower than the exponential complexity of exhaustive search. Therefore, the proposed algorithm is suitable for large-scale RIS systems.
**Algorithm 1:** Successive refinement algorithmInitialize: $\Theta =\Theta^{\left(0\right)}$ Setup: k=0, $R^{\left(0\right)}=\log_{2}\left(1+\frac{P\|h_{d}+H_{br}\Theta h_{rv}{\|}^{2}}{N_{0}}\right)$ While $|R^{\left(k\right)}-R^{\left(k-1\right)}|>\epsilon$ do                  for n=0 to N do
$\theta_{n}^{*}=\arg\min_{\theta \in F}|\theta -k_{n}|$
k=k+1
$R^{\left(k\right)}=\log_{2}\left(1+\frac{P\|h_{d}+H_{br}\Theta h_{rv}{\|}^{2}}{N_{0}}\right)$ end end

### 3.1. Group-Based RIS Phase Optimization

Dividing the RIS array into several subgroups and optimizing the common phase shift in each subgroup is an effective approach to reducing the channel-estimation overhead. After grouping, all reflecting elements in each subgroup are treated as a single equivalent element. Therefore, only the equivalent channel of each subgroup needs to be estimated rather than the channels of all individual reflecting elements within the subgroup.

Assume that the RIS consists of N total reflecting elements with a phase-shift vector of $\theta ={\left[\theta_{1},\ldots ,\theta_{N}\right]}^{T}$, where $\theta_{n}\in F$. In the grouping strategy, we divide these N components into G disjoint subgroups $N_{1},\ldots ,N_{G}$, where $N=\overset{G}{\underset{g=1}{\cup }}N_{g}=\{1,\ldots ,N\}$ and $N_{i}\cap N_{j}=\emptyset$ (when i≠j). Make $N_{g}=|N_{g}|$ for the number of elements in the first g child group, the $N=\sum_{g=1}^{G}N_{g}$.

The key principle of grouping is that all reflecting elements within the same subgroup share the same phase-shift value. We define the G child-group phase-shift $\phi ={\left[\phi_{1},\ldots ,\phi_{G}\right]}^{T}$, where $\phi_{g}\in F$. Therefore, each element $\theta_{n}$ in the original N dimensional phase shift vector θ is constrained as

(23) $$\theta_{n}=\phi_{g},\;\forall n\in N_{g}$$

Accordingly, the optimization variables in Equation (20) are reduced from N independent element-level phase shifts $\theta_{1},\ldots \theta_{N}$ to G independent subgroup-level phase shifts $\phi_{1},\ldots \phi_{G}$. Therefore, the optimization problem in Equation (20) can be reformulated as

(24) $$\underset{\phi \in F^{G}}{\max}\;R=\log_{2}(1+\mathrm{SNR}(\Theta (\phi )))$$

(25) $$s.t.\;\theta_{n}=\phi_{g},\;\forall n\in N_{g},\;g=1,\ldots ,G$$

**Figure 3:**
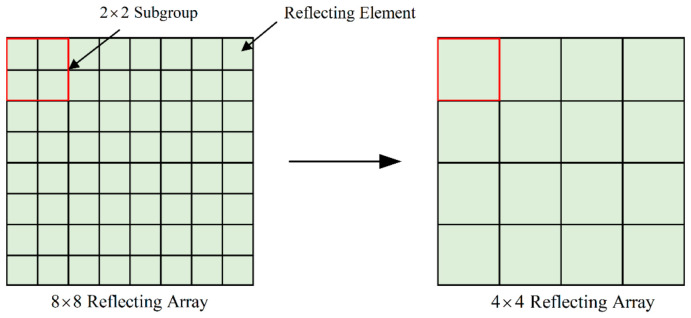
Schematic diagram of reflection array divided into subgroups.

Figure 3 illustrates the grouping procedure for an 8×8 RIS array. The 8×8 reflecting array is divided into subgroups, each consisting of 2×2 reflecting elements. Each subgroup is then treated as a single equivalent reflecting element. Thus, the original array can be represented as an equivalent 4×4 reflecting array. Phase optimization is performed on the equivalent 4×4 array, thereby reducing both the channel-estimation overhead and the computational complexity of the successive refinement algorithm. The key principle is that after subgroup-level phase optimization, all reflecting elements within the same subgroup are assigned the same subgroup-level phase shift.

Furthermore, the selection of subgroup size essentially determines the trade-off relationship between beamforming accuracy and computational cost. The subgroup of 1×1 is equivalent to directly using the complete channel state information without grouping, which can maximize spatial resolution and achievable rate, but it will bring excessive channel-estimation overhead and optimization complexity. The subgroup of 4×4 can significantly reduce the number of optimization variables and computational overhead, but at the cost of reduced phase-alignment accuracy and decreased achievable rate. Therefore, systematically analyzing these different subgroup sizes is crucial for balancing system performance and computational efficiency, allowing readers to select the appropriate subgroup size based on specific deployment constraints.

### 3.2. Position-Based Optimization of RIS Phase

The reflecting elements of the RIS receive the incident signal and reradiate it toward the desired directions by adjusting their phase shifts. For an RIS-assisted cascaded propagation path [31], the effective path-loss factor in the linear scale is modeled as the product of the path-loss factors of the constituent links. Therefore, the direct MS-to-BS transmission generally provides a stronger received signal. However, when the direct link is blocked or severely attenuated, the RIS-assisted link can maintain a relatively high received-signal strength under favorable LoS propagation conditions. When the direct MS-to-BS link is blocked, the RIS-assisted LoS path becomes critical for reliable signal reception at the BS. Accordingly, the RIS phase shifts can be optimized according to the geometric relationship among the MS, RIS, and BS. In high-mobility vehicular scenarios, the LoS component typically dominates the millimeter-wave (mmWave) channel. Unlike conventional approaches that estimate the channel gain associated with each RIS element, the proposed method directly reconstructs the LoS-dominated channel and optimizes the RIS phase shifts by exploiting position-related geometric information. The position-based design reconstructs only the deterministic LoS components from geometry; the stochastic NLoS components are retained in the composite channel used for performance evaluation and are not inferred solely from position information.

Let the coordinates of the BS, the center of the RIS, and the vehicle be denoted as $p_{B}=(x_{B},y_{B},z_{B})$, $p_{R}=(x_{R},y_{R},z_{R})$, and $p_{V}=(x_{V},y_{V},z_{V})$, respectively. As shown in Figure 2, the geometric relationship allows us to calculate the distance and angles required to reconstruct the channel.

The RIS-to-BS distance $d_{br}$ and the vehicle-to-RIS distance $d_{rv}$ can be expressed as follows:

(26) $$d_{br}=\|p_{B}-p_{R}\|,\;d_{rv}=\|p_{R}-p_{V}\|$$

Based on the coordinates, the elevation angle θ and azimuth angle ϕ for the BS-to-RIS link $\left(\theta_{br},\phi_{br}\right)$ and RIS-to-vehicle link ($\left(\theta_{rv},\phi_{rv}\right)$) can be derived. For instance, the angles at the RIS side are given by

(27) $$\theta =\arcsin\left(\frac{z_{target}-z_{R}}{d}\right),\;\phi =\arctan\left(\frac{y_{target}-y_{R}}{x_{target}-x_{R}}\right)$$

Let ${\bar{h}}_{d,\mathrm{LoS}}$ denote the reconstructed LoS component of the direct MS-to-BS channel. Let ${\bar{H}}_{br,\mathrm{LoS}}$ and ${\bar{h}}_{rv,\mathrm{LoS}}$ denote the reconstructed LoS components of the RIS-to-BS and vehicle-to-RIS channels, respectively. Furthermore, let ${\bar{h}}_{br,\mathrm{LoS},n}$ denote the n-th column of ${\bar{H}}_{br,\mathrm{LoS}}$, and let ${\bar{h}}_{rv,\mathrm{LoS},n}$ denote the n-th entry of ${\bar{h}}_{rv,\mathrm{LoS}}$:

(28) $$\begin{array}{l} c_{d}=w^{H}{\bar{h}}_{d,\mathrm{LoS}},\\ c_{n}=w^{H}{\bar{h}}_{br,\mathrm{LoS},n}{\bar{h}}_{rv,\mathrm{LoS},n},\;n=1,\ldots ,N. \end{array}$$

where $c_{d}$ represents the complex coefficient of the direct LoS component after receive combining and $c_{n}$ represents the complex coefficient of the reflected LoS component associated with the n-th RIS element.

To obtain constructive combining, each RIS phase must compensate for the phase difference between the corresponding reflected path and the direct LoS path. The continuous phase and its discrete projection are therefore given by

(29) $$\begin{array}{l} \theta_{n}^{\mathrm{cont}}={\left[\phi_{\mathrm{ref}}-\arg(c_{n})\right]}_{2\pi },\\ \theta_{n}^{*}=Q_{F}\left(\theta_{n}^{\mathrm{cont}}\right),\;n=1,\ldots ,N. \end{array}$$

According to the system model in Equation (9), the RIS array response vector $\alpha_{\mathrm{RIS}}(\theta ,\phi )$ is determined by these angles. Accordingly, for n=1,…,N, the cascaded channel coefficient associated with the n-th RIS element can be reconstructed as follows:

(30) $$H_{br}=\sqrt{PL_{\mathrm{LOS}}}e^{-j\frac{2\pi }{\lambda }(d_{br}+d_{rv})}{\left[\alpha_{\mathrm{RIS}}(\theta_{br},\phi_{br})\right]}_{n}{\left[\alpha_{\mathrm{RIS}}(\theta_{rv},\phi_{rv})\right]}_{n}$$

where ${\left[\cdot \right]}_{n}$ denotes the n-th entry of the vector.

To maximize the achievable rate in (19), the phase shift $\theta_{n}$ of the n-th RIS element should be configured to align the phase of the RIS-reflected vehicular signal with that of the direct signal received at the BS, thereby enabling constructive signal combining. The optimal continuous phase shift $\theta_{n}^{opt}$ is given by

(31) $$\theta_{n}^{opt}=-∠\left({\left[\alpha_{\mathrm{RIS}}(\theta_{br},\phi_{br})\right]}_{n}\cdot {\left[\alpha_{\mathrm{RIS}}(\theta_{rv},\phi_{rv})\right]}_{n}\right)$$

The proposed position-based method differs from existing optimization approaches in the following key aspects:

Overhead Reduction: Traditional element-wise channel-estimation methods, such as LS and MMSE estimators, require pilot overhead that scales with the number of RIS elements N. In contrast, the proposed position-based method decouples the pilot overhead from N. It requires only the coordinate or angular information of the MS, RIS, and BS, thereby substantially reducing the pilot overhead.

Computational Complexity: Conventional optimization methods such as semidefinite relaxation (SDR) and alternating optimization (AO) typically require complex matrix operations and iterative updates until convergence. In contrast, the proposed position-based method relies only on closed-form geometric calculations and simple algebraic operations, thereby substantially reducing the computational complexity.

### 3.3. Other Phase-Optimization Algorithms

In addition to the two RIS phase-optimization algorithms presented above, three commonly used phase-optimization algorithms are introduced in this section: semidefinite relaxation (SDR), alternating optimization (AO), and compressive sensing (CS).

(1) The core idea of the compressive-sensing (CS) method is to exploit channel sparsity for efficient channel reconstruction and subsequent phase-shift optimization. In RIS-assisted scenarios, the channel matrix often exhibits sparsity in the angular, delay, or Doppler domains. CS can reconstruct the high-dimensional channel from a limited number of measurements, thereby reducing the channel-estimation overhead [32].
(2) The core idea of the semidefinite relaxation (SDR) method is to convert the nonconvex phase-shift optimization problem into a semidefinite programming (SDP) problem via matrix lifting and relaxation of the resulting rank-one constraint. A feasible phase-shift solution is then recovered through Gaussian randomization or projection onto the feasible set [19].
(3) The alternating optimization (AO) method decomposes a complex joint optimization problem into several tractable subproblems. Each variable block is then optimized in turn while the remaining variables are fixed. This process is repeated until convergence is achieved [20]. A comparison of the time complexities and main computational operations of the considered optimization algorithms is presented in Table 1.

## 4. Analysis of Simulation Results

### 4.1. Simulation Environment

**Figure 4:**
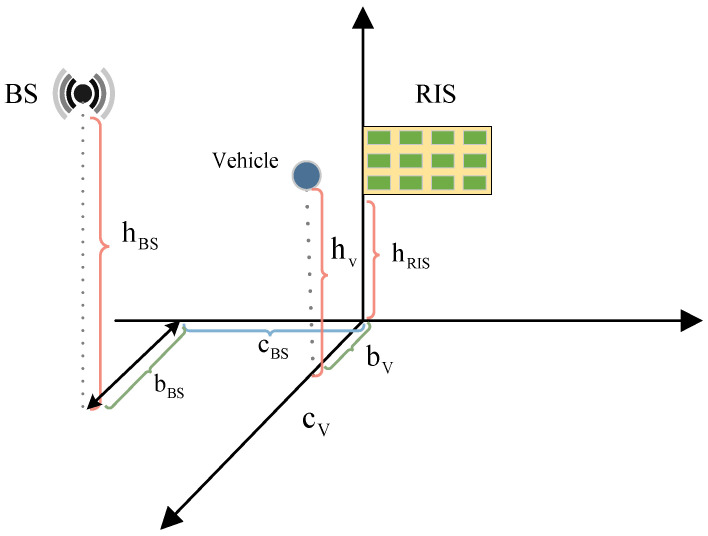
Schematic diagram showing equipment positions in simulation results.

Numerical simulations are conducted using MATLAB R2024a to validate the proposed algorithms and evaluate the performance of RIS-assisted vehicular communication. A millimeter-wave carrier frequency of $f_{c}=28\mathrm{GHz}$ is adopted in the simulations. The positions of the BS, RIS array, and MS are shown in Figure 4. The BS is equipped with an eight-element antenna array. The RIS employs a planar reflecting array with 16 × 16 reflecting elements. The MS is equipped with a single antenna. The RIS is mounted parallel to the y-z plane, with its center height set to $h_{\mathrm{RIS}}=2\;m$. The horizontal projection of the BS is located on the x-y plane. The BS height is set to $h_{\mathrm{BS}}=4\;m$, and its horizontal distances from the y and x axes are $b_{\mathrm{BS}}=20\;m$ and $c_{\mathrm{BS}}=10\;m$, respectively. The MS antenna is placed on the x-z plane, with its projection lying on the x-axis. Its height and horizontal distance from the y-axis are $h_{v}=1\;m$ and $b_{v}=1\;m$, respectively. The detailed simulation parameters are listed in Table 2.

All channels in the simulations are modeled as Rician fading channels. The 3GPP TR 38.901 UMi street-canyon path-loss model is adopted to characterize large-scale path loss. We use $\beta_{r}=2$ for the RIS-BS link, $\beta_{\nu }=1$ for the vehicular-RIS link, and $\beta_{d}=\infty$ for the direct link. Meanwhile, we conducted experimental simulations for the CSI of RIS in the simulation.

**Figure 5:**
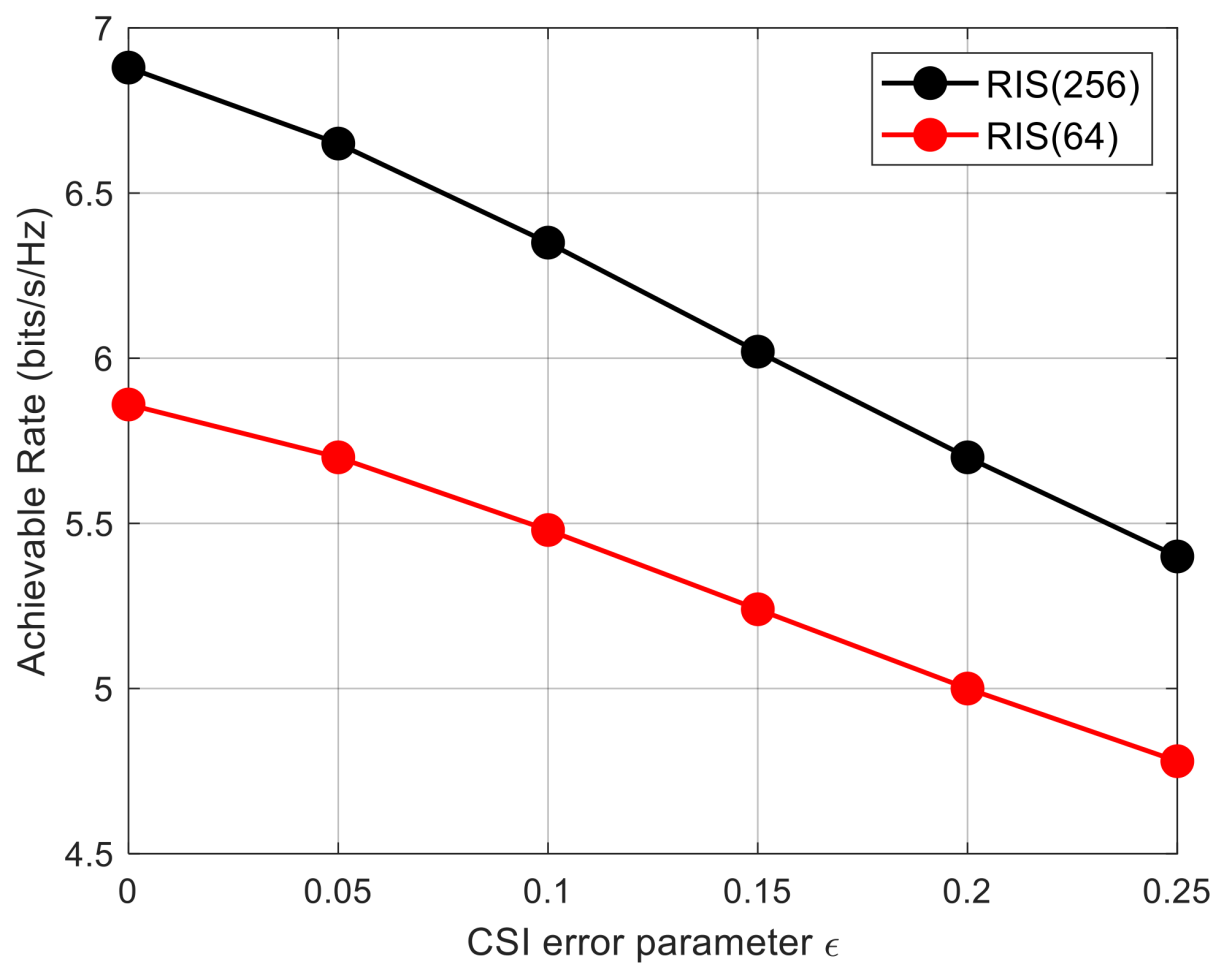
Comparison of achievable rates for different channel error parameters.

Figure 5 shows the relationship between the CSI error parameters and the achievable rate. In the simulation, the transmission power was fixed at 30 dBm. From the simulation results, it can be seen that as the CSI error parameters increase, the achievable rate gradually decreases. Moreover, the more RIS reflection units there are, the more significant the decrease in the achievable rate when there is CSI error. When the CSI error parameters were set to 0.25, the achievable rate obtained by using 256 RIS reflection units dropped to 5.42 bits $s^{-1}{Hz}^{-1}$. This result indicates that accurate CSI is crucial for maintaining system performance. Therefore, in the simulation, to ensure fair and uniform CSI, all channels related to RIS were assumed to have perfect CSI.

**Figure 6:**
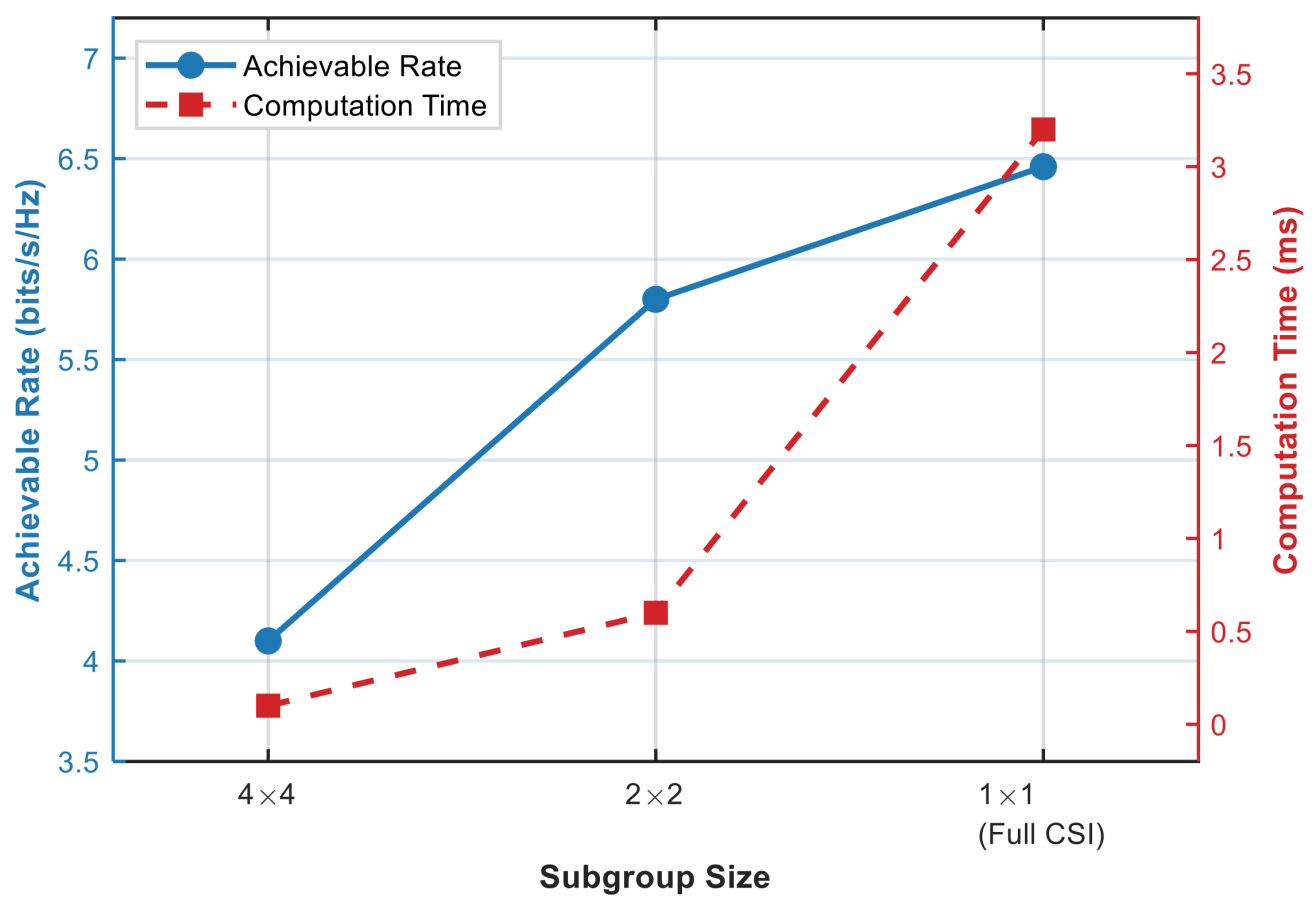
The achievable rate and computing time under different subgroups (256-element RIS, transmission power = 30 dBm).

Figure 6 shows the relationship between achievable rate and computational complexity for different subgroup sizes. It can be observed that when using the 1×1 configuration (full CSI), the upper limit of the achievable rate is approximately 6.5 bits $s^{-1}{Hz}^{-1}$, but the computational time is the longest, taking about 3.2 ms. By configuring a 2×2 subgroup structure, the computational time is approximately 0.6 ms, but the rate slightly decreases by about 0.7 bits $s^{-1}{Hz}^{-1}$. The 4×4 subgroup further reduces the execution time to 0.1 ms, but due to significant loss of spatial degrees of freedom, its rate significantly decreases. This quantitative assessment provides clear guidance for readers, enabling them to adaptively select the optimal clustering granularity based on actual hardware limitations and real-time requirements.

### 4.2. Analysis of Simulation Results

**Figure 7:**
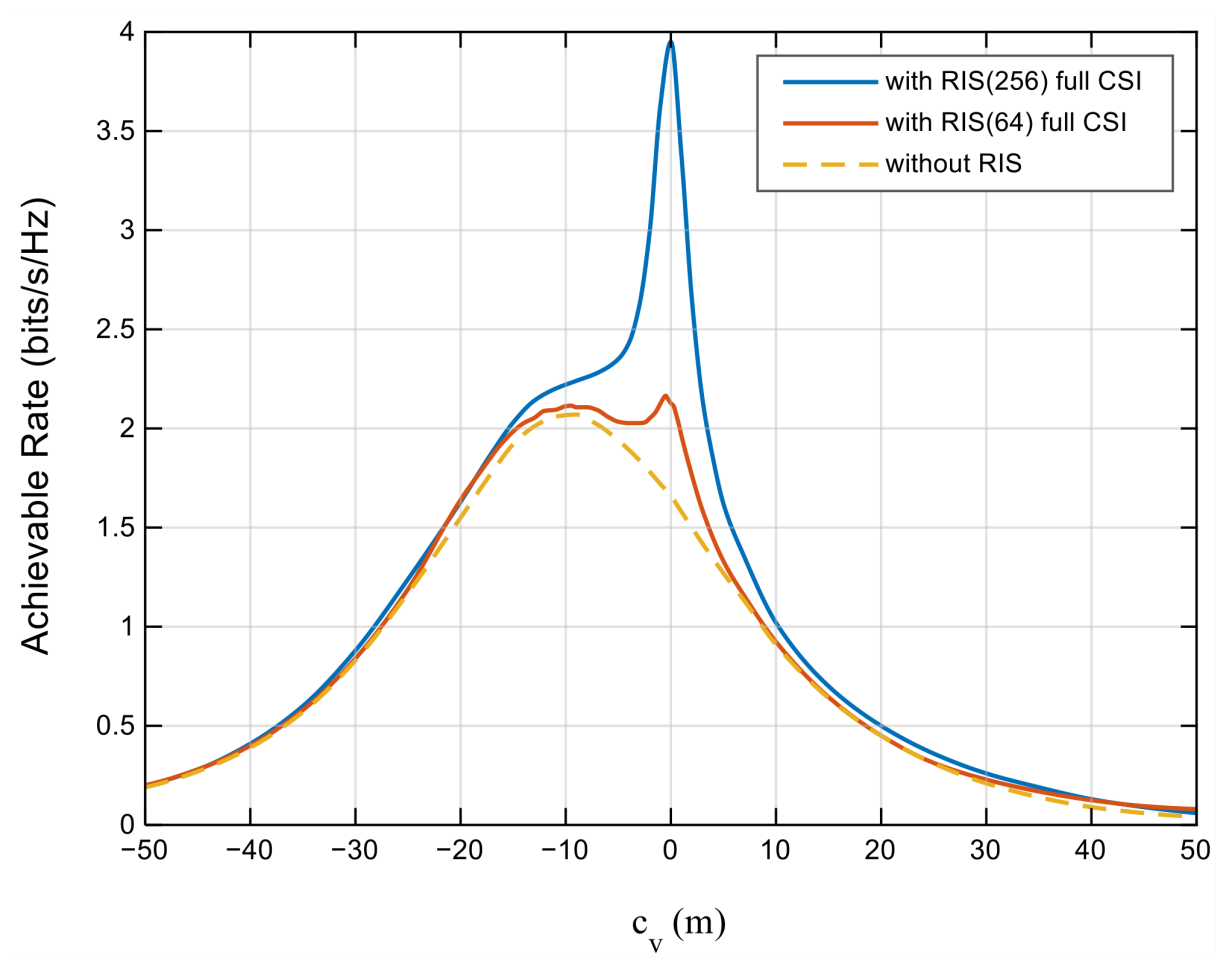
Rate as a function of the position of the vehicle for 16 × 16 and 8 × 8 RIS reflection arrays and for no RIS.

Figure 7 illustrates the variation in the achievable rate with respect to the MS position. In the simulations, RIS arrays with 16×16 and 8×8 reflecting elements are considered. The results show that when the MS is closest to the RIS at $c_{v}=0$, the RIS significantly improves the achievable rate. Specifically, the RIS with 256 reflecting elements achieves a maximum achievable rate of 4 bits $s^{-1}{Hz}^{-1}$. However, the achievable-rate gain decreases as the MS moves away from the RIS. This trend is mainly attributed to the reduced MS-to-RIS path loss near the RIS, which leads to a stronger RIS-reflected signal.

To compare the group-based and position-based designs in terms of the achievable rate, the MS is fixed at the position closest to the RIS, corresponding to $c_{v}=0$. The achievable rates obtained with RIS arrays consisting of 16×16, 8×8, and 4×4 reflecting elements are compared as the transmit power varies.

**Figure 8:**
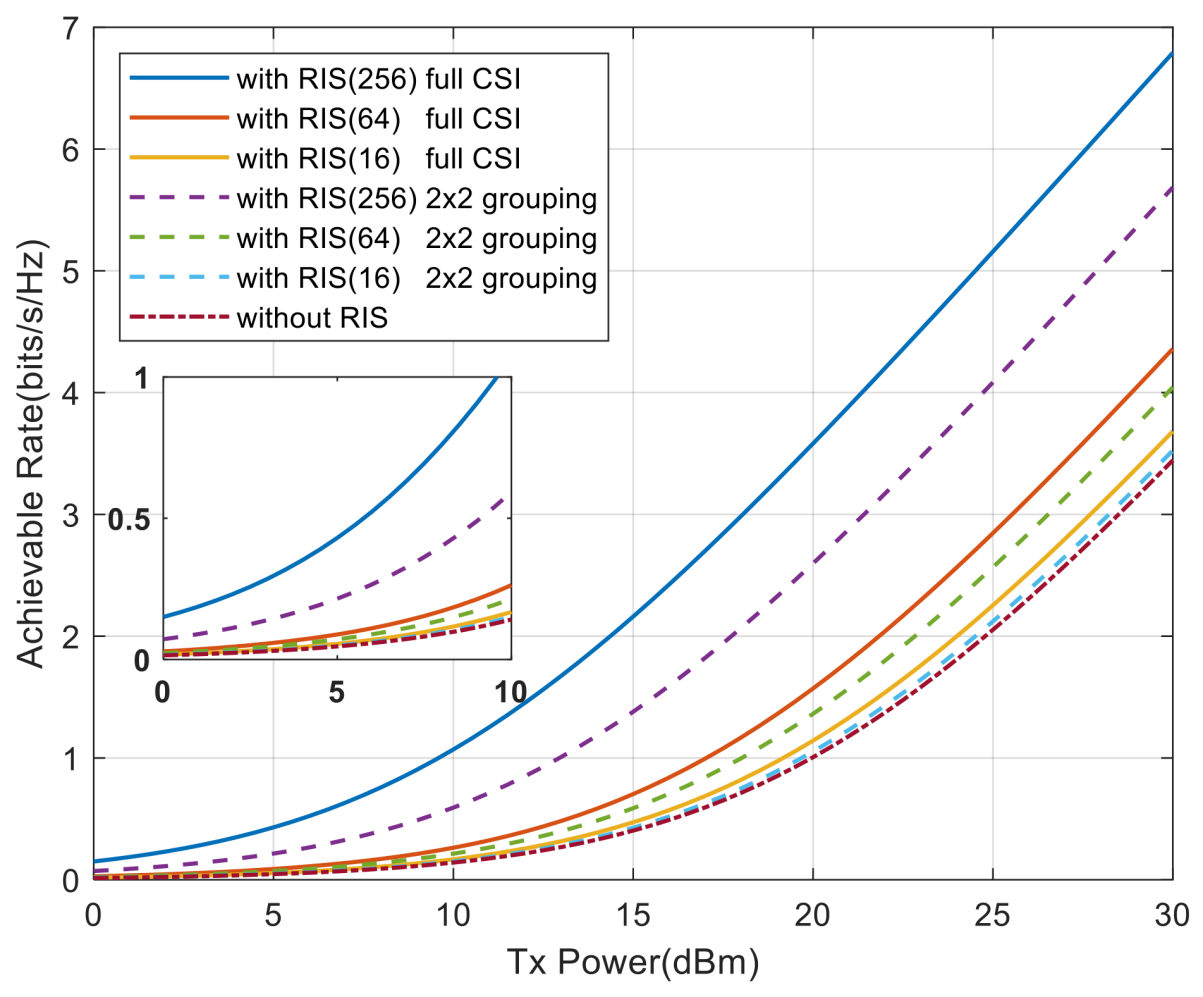
Rate as a function of transmission power for 16 × 16, 8 × 8, and 4 × 4 RIS arrays with either full CSI or 2 × 2 grouping. Also shown for comparison is the scenario with no RIS.

Figure 8 presents a comparison of the achievable rates under different transmit-power levels for the group-based design. The simulation results show that the group-based design causes achievable-rate degradation. The extent of this degradation depends on the size of the RIS array. As the RIS array size increases, the achievable-rate degradation becomes more pronounced. Nevertheless, all RIS-assisted cases yield higher achievable rates than the non-RIS-assisted baseline. When 2×2 grouping is applied to the 16×16 and 8×8 RIS arrays, their effective control dimensions are reduced to those of 8×8 and 4×4 arrays for passive beamforming. The grouping-based configuration provides a higher achievable rate than the 8×8 and 4×4 reflecting-array configurations with perfect channel state information (CSI). For example, when the transmit power is 30 dBm, the achievable rate of the 16×16 reflecting array with 2×2 element grouping is approximately 1 bits $s^{-1}{Hz}^{-1}$ higher than that of the 8 × 8 reflecting array with perfect CSI. These results demonstrate that element grouping provides an effective phase-optimization strategy for large-scale reflecting arrays while reducing the overhead associated with channel estimation.

**Figure 9:**
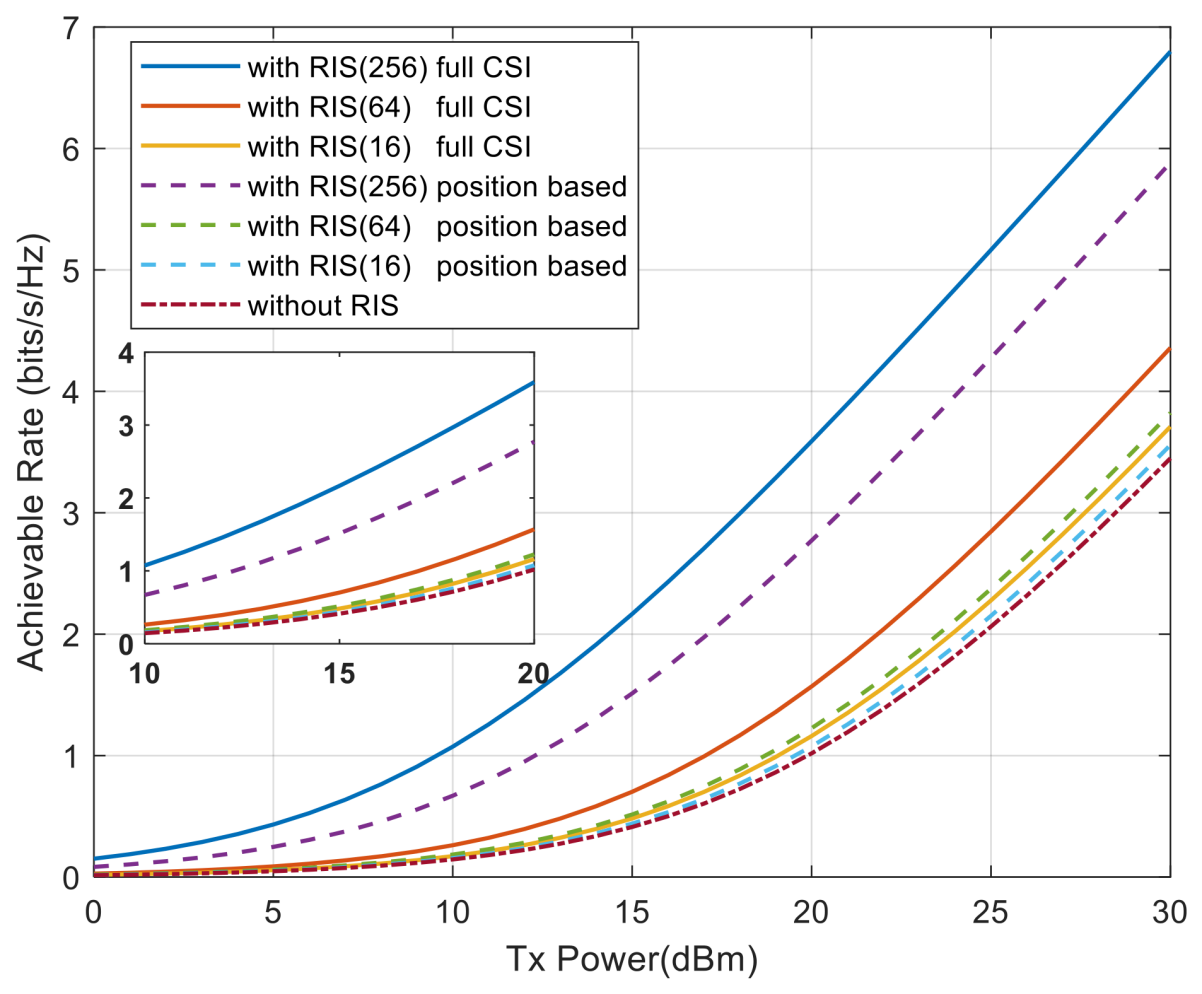
Rate as a function of transmission power for 16 × 16, 8 × 8, and 4 × 4 RIS arrays with either full CSI or based on position. Also shown for comparison is the scenario with no RIS.

Figure 9 compares the achievable rates of the position-based phase-optimization scheme with those of the perfect-CSI benchmark and the non-RIS-assisted baseline at different transmit powers. Although the position-based scheme exhibits a moderate rate loss relative to the perfect-CSI benchmark, the position-based 16×16 reflecting-array configuration substantially improves the achievable rate over the non-RIS-assisted baseline. However, the achievable-rate gains obtained by the 8×8 and 4×4 reflecting-array configurations are relatively limited. This result can be attributed to the larger effective aperture provided by a larger reflecting array, which enables the LoS component to be exploited more effectively for beamforming. The smaller effective apertures of the 8×8 and 4×4 reflecting arrays restrict the beamforming gain obtained from the LoS component, resulting in less pronounced performance improvement. These results indicate that the position-based beamforming scheme is more suitable for large-scale reflecting arrays, as it can reduce the channel-estimation overhead while improving the achievable rate. By contrast, its achievable-rate gain is limited for small-scale reflecting arrays.

**Figure 10:**
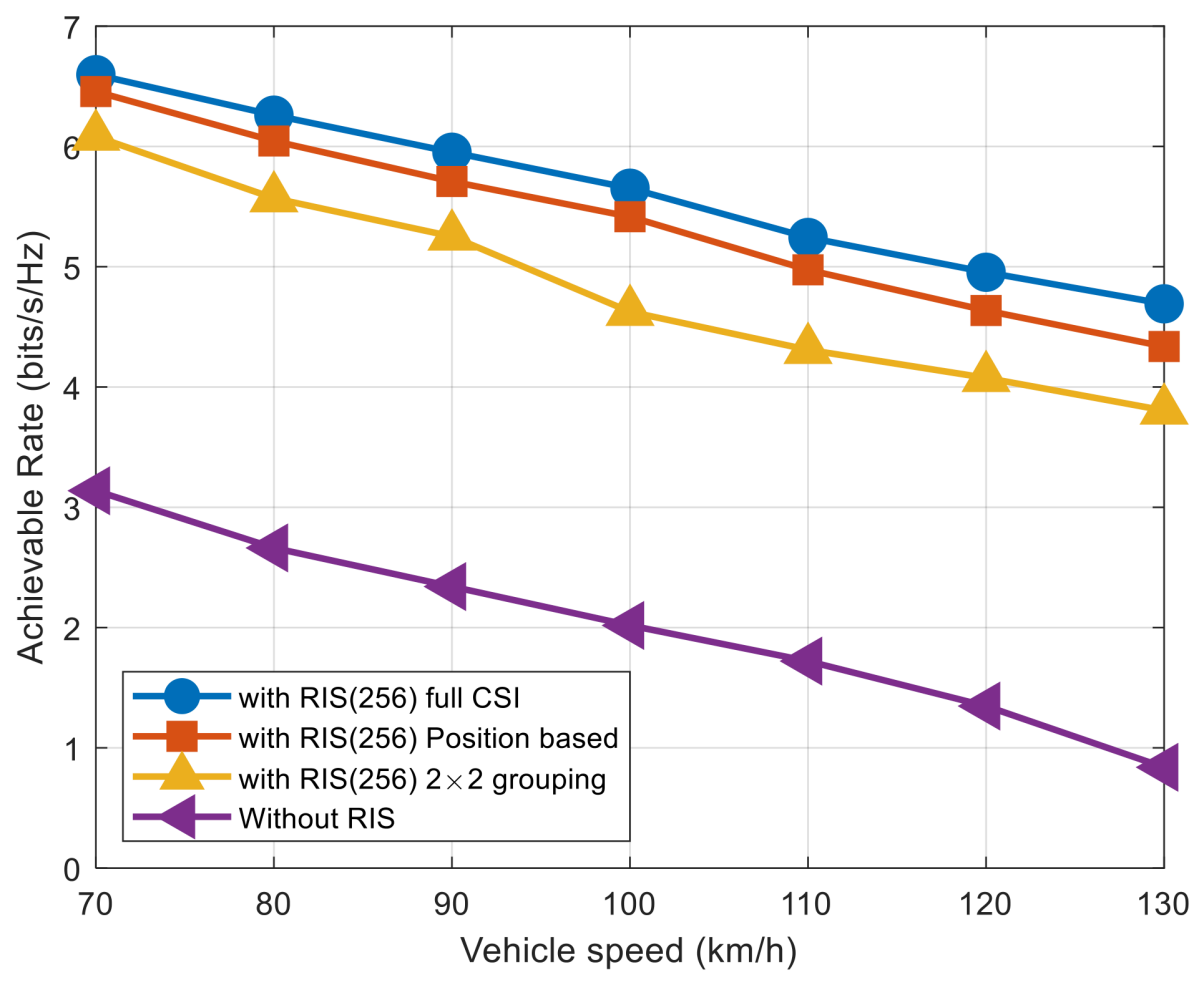
Achievable rate at different vehicle speeds.

Figure 10 presents how the achievable rate varies with vehicle speed. The achievable rates of all considered schemes decrease as the vehicle speed increases. The perfect-CSI benchmark represents the performance upper bound and reaches nearly 7 bits $s^{-1}{Hz}^{-1}$ at a vehicle speed of 70 km/h, thereby substantially outperforming the non-RIS-assisted baseline. Furthermore, the position-based scheme achieves a higher achievable rate than the 2×2 element-grouping scheme. This result indicates that the position-based and grouping-based RIS configurations are effective and robust design choices that can provide achievable-rate gains close to those of the perfect-CSI benchmark even under high-mobility conditions.

**Figure 11:**
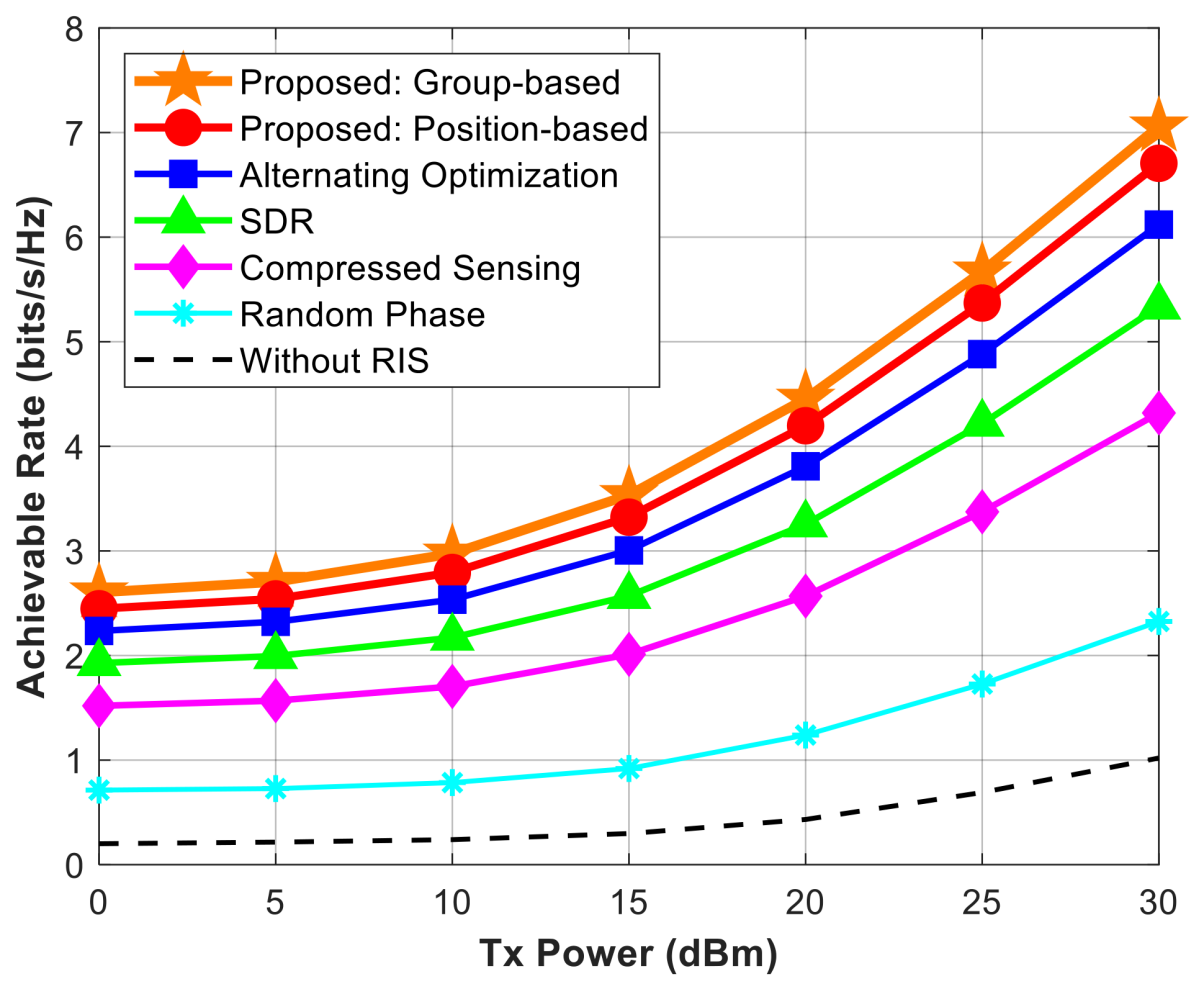
Performance comparison of RIS phase-optimization methods.

Figure 11 presents the achievable rates of different RIS phase-optimization methods at different transmit powers. The proposed position-based and grouping-based methods outperform the other considered methods. In particular, the position-based method reaches an achievable rate of approximately 7 bits $s^{-1}{Hz}^{-1}$ at a transmit power of 30 dBm. The alternating-optimization and SDR methods rank second, reaching achievable rates of approximately 6 bits $s^{-1}{Hz}^{-1}$. The compressed-sensing and random-phase methods reach approximately 5 bits $s^{-1}{Hz}^{-1}$ and 4 bits $s^{-1}{Hz}^{-1}$, respectively. The non-RIS-assisted baseline consistently yields the lowest achievable rate, indicating that RIS phase optimization provides a clear performance advantage.

**Figure 12:**
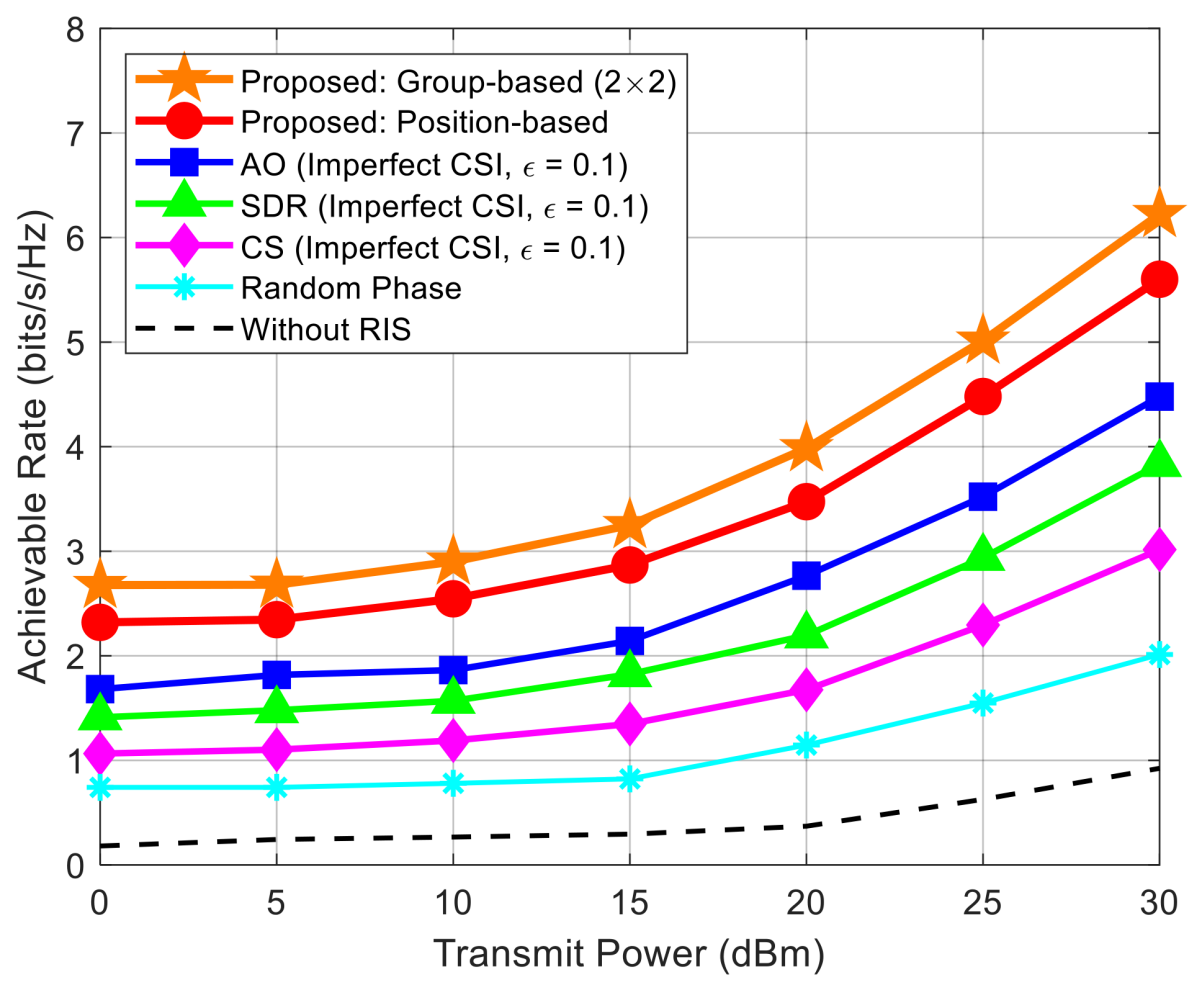
Performance comparison under imperfect CSI conditions.

Figure 12 presents the simulation comparison results of the system’s achievable rate as the transmission power varies when there is imperfect channel state information (imperfect CSI, ϵ=0.1). From the simulation results, it can be seen that the achievable rates of all schemes increase with the increase in transmission power. Within the entire transmission power range, the performance of the algorithm based on group optimization (group-based, 2 × 2) proposed in this paper and the algorithm based on location assistance (position-based) is significantly superior to other traditional benchmark schemes. Among them, the algorithm based on group optimization has the best performance, achieving a rate of approximately 6.2 bits $s^{-1}{Hz}^{-1}$ when the transmission power is 30 dBm. It is worth noting that the traditional alternating optimization (AO), semidefinite relaxation (SDR), and compressive-sensing (CS) algorithms, due to their heavy reliance on instantaneous channel estimation, exhibit significant performance degradation under the interference of errors caused by imperfect CSI. In contrast, the two schemes proposed in this paper demonstrate better noise resistance robustness.

**Figure 13:**
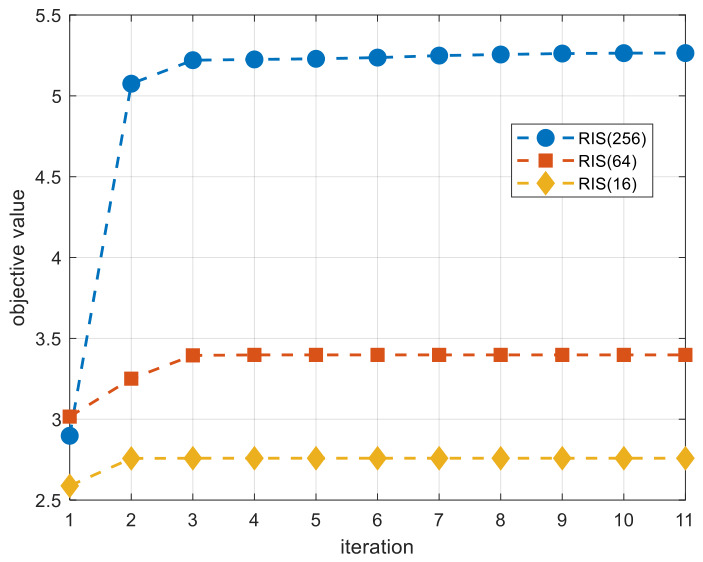
Convergence performance of successive refinement algorithms for different RIS elements.

Figure 13 shows the convergence behavior of the successive refinement algorithm. The 4×4 reflecting-array configuration reaches convergence after only two iterations, whereas the 8×8 and 16×16 reflecting-array configurations require three iterations. This result indicates that the successive refinement algorithm maintains rapid convergence even as the number of reflecting elements increases. In addition, the objective value increases as the number of RIS reflecting elements increases. This is mainly because a larger RIS array provides more spatial degrees of freedom and enables more effective phase refinement while maintaining rapid convergence.

Table 3 compares the average convergence time of the successive refinement algorithm for different numbers of RIS reflecting elements. In the simulation, the algorithmic error threshold is set to 10 ^−3^ as the convergence criterion. The maximum number of inner iterations is set to 10, and 100 outer Monte Carlo trials are conducted.

The algorithm was executed on a workstation equipped with an Intel Core i5-13490F CPU, 32 GB of RAM and an NVIDIA GeForce RTX 5060 Ti GPU. The software environment was MATLAB R2024a. The average convergence time increases as the number of RIS reflecting elements increases. Nevertheless, it remains below 0.01 s in all cases. This result indicates that the successive refinement algorithm maintains rapid convergence even for large-scale RIS arrays.

**Figure 14:**
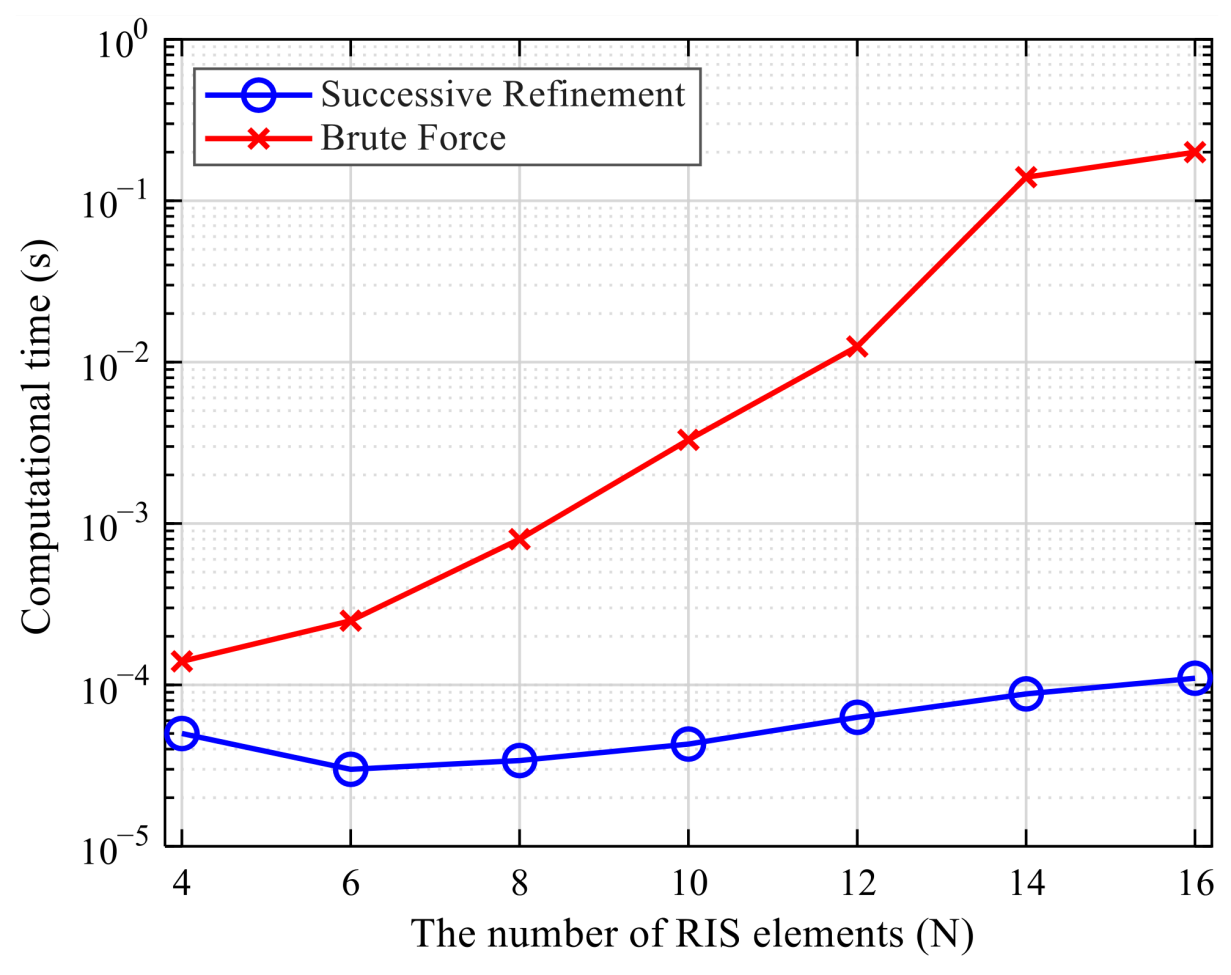
Computation time for different numbers of RIS elements.

Figure 14 compares the computational time of the brute-force method and the successive refinement algorithm. The brute-force method is unsuitable for medium- and large-scale RIS arrays because its computational complexity increases exponentially with the number of reflecting elements. Therefore, the comparison is restricted to small-scale RIS arrays. The simulation results show that the computational time of the successive refinement algorithm is substantially lower than that of the brute-force method. As the number of RIS reflecting elements increases, the computational-time gap between the two methods widens. The successive refinement algorithm requires only 0.0001 s for a 16-element RIS.

**Figure 15:**
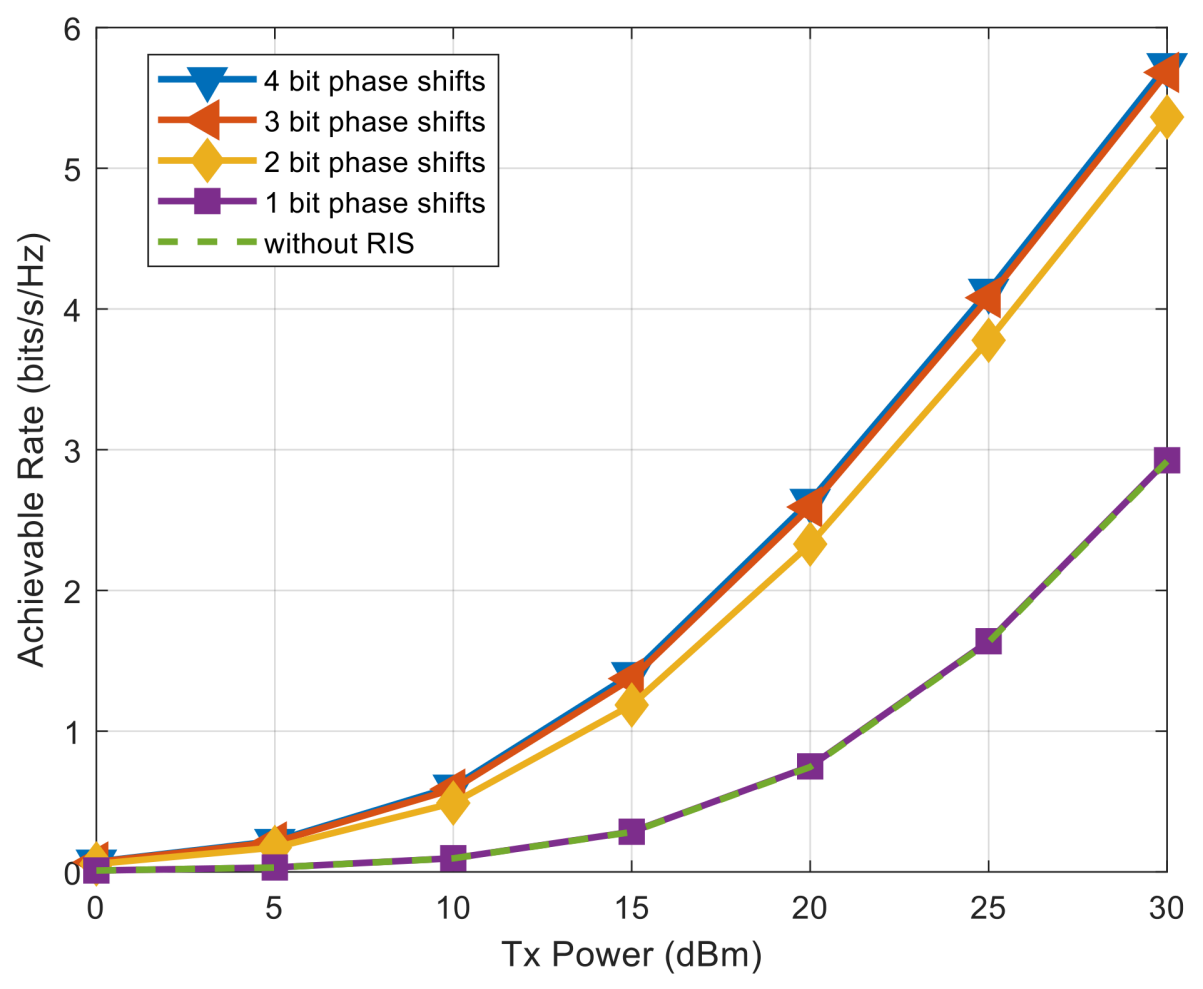
Rate as a function of transmission power for different discretized phase shifts.

Figure 15 presents the achievable rates under different quantized phase-shift resolutions. The simulation results show that the 1-bit phase-shift configuration provides performance comparable to that of the non-RIS-assisted baseline. Increasing the phase-shift resolution to 2 bits substantially improves the achievable rate. A further increase to 3 bits yields only a slight additional gain, which is less pronounced than the gain obtained when the resolution is increased from 1 bit to 2 bits. Increasing the resolution to 4 bits provides almost no additional performance improvement. Therefore, the results indicate that 2-bit quantized phase-shift control is sufficient to improve the achievable rate of the RIS-assisted system. This observation is consistent with practical RIS implementations, which usually support only a finite number of phase-shift bits.

## 5. Conclusions

To reduce the high channel-estimation overhead in RIS-assisted vehicular communication scenarios, two phase-optimization methods are proposed in this paper. The first is a grouping-based phase-optimization method, in which the RIS array is partitioned into multiple subgroups. The phase shift in each subgroup is then optimized to effectively reduce the channel-estimation overhead. The second is a position-based phase-optimization method. In this method, the channel matrix is reconstructed using device-location information to further reduce the channel-estimation overhead. A successive refinement algorithm is introduced to reduce the high computational complexity associated with phase optimization in the two methods described above. The proposed algorithm simplifies the channel-gain expression and reduces the complexity of the phase-optimization process. The simulation results show that the proposed grouping-based and position-based RIS phase-optimization methods provide higher achievable rates than the other considered phase-optimization algorithms. The successive refinement algorithm also exhibits rapid convergence for the considered large-scale RIS arrays. For a 256-element RIS, convergence is achieved within three iterations. The average convergence time remains below 0.01 s. This paper mainly provides a preliminary theoretical reference for RIS phase optimization. However, the current research is limited to single-antenna setups and relies on simplified hardware assumptions. To address these limitations, it is important for us to extend the proposed framework to multi-antenna systems and incorporate the actual non-ideal characteristics of the hardware in our future research.

## Figures and Tables

**Table 1:** Time complexity comparison of different optimization algorithms.

Algorithm	Time Complexity	Main Computational Operations
Group Optimization	O(G⋅L⋅M⋅N)	Matrix multiplication
Position Optimization	O(N)	Geometric calculation
Compressed Sensing	O(K⋅M⋅N)	Inner product, least squares
SDR Method	$O(N^{3})$	Eigenvalue decomposition
Alternating Optimization	O(T⋅N⋅L⋅M⋅N)	Iterative optimization

Notation: N is the total number of RIS reflection elements; M is the number of BS antennas; L is the discrete phase series; G is the number of groups ($G=N/g^{2}$, g is the group size); K is the compressive-sensing sparsity; T is the number of alternating optimization iterations.

**Table 2:** Simulation parameters.

Parameter Name (Unit)	Parameter Value
Carrier Frequency (GHz)	$f_{c}=28\mathrm{GHz}$
BS Transmit Power Range (dBm)	$P_{t}=0\sim 30\mathrm{dBm}$
Noise Power (dBm)	$\sigma^{2}=-60\mathrm{dBm}$
Number of BS Antennas	$N_{\mathrm{BS}}=8$
Number of MS Antennas	$N_{\mathrm{MS}}=1$
Number of RIS Reflecting Elements	N=16/64/256
Antenna and Reflecting Element Spacing	$d=\frac{\lambda }{2}$
RIS Subarray Size	$K_{r}\times K_{c}=2\times 2$
Number of Discrete Phase-Shift Levels	L=8
BS Position Coordinates (m)	$\left(x_{B},y_{B},z_{B}\right)=\left(20,-10,2\right)$
RIS Position Coordinates (m)	$\left(x_{R},y_{R},z_{R}\right)=\left(0,0,1\right)$
Initial MS Position (m)	$\left(x_{V},y_{V},z_{V}\right)=\left(1.5,0,1\right)$
Channel Fading Model	Rician Fading Model

**Table 3:** Algorithm running time under different quantities of RIS reflecting elements.

RIS Reflecting Elements	Time (s)
256	0.0032
64	0.0006
16	0.0001

## Data Availability

The data supporting the findings of this study are available from the corresponding author upon reasonable request.

## Abbreviations

The following abbreviations are used in this manuscript:

RIS	Reconfigurable intelligent surface
BS	Base station
CSI	Channel state information
ITS	Intelligent transportation system
V2V	Vehicle-to-vehicle
V2I	Vehicle-to-infrastructure
V2P	Vehicle-to-pedestrian
V2N	Vehicle-to-network
V2X	Vehicle-to-everything
5G	Fifth-generation wireless network
6G	Sixth-generation wireless network
mmWave	Millimeter wave
3GPP	Third-Generation Partnership Project
LOS	Line-of-sight
SNR	Signal-to-noise ratio
LS	Least squares
SDR	Semidefinite relaxation
SDP	Semidefinite programming
AO	Alternating optimization
CS	Compressive sensing
CPU	Central processing unit
GPU	Graphics processing unit

## Appendix A

**Proof of Equation (21).** The left side of Equation (21) is the square of a vector norm, which is equivalent to the product of the vector and its conjugate transpose.

(A1) $$\left\|h_{d}+H_{br}\Theta h_{rv}{\|}^{2}={\left(h_{d}+H_{br}\Theta h_{rv}\right)}^{H}(h_{d}+H_{br}\Theta h_{rv}\right)$$

where Θ is the RIS reflection matrix and v is the complex vector of RIS reflection phase shift; therefore, we can conclude that

(A2) $$\Theta h_{rv}=\mathrm{diag}(v)h_{rv}=h_{rv}⊙v$$

where ⊙ is the element-wise product. Further, we obtain

(A3) $$H_{br}\Theta h_{rv}=H_{br}(\mathrm{diag}(v)h_{rv})=(H_{br}\mathrm{diag}(h_{rv}))v=\Phi v$$

Substitute Formula (26) into (22) and expand the expression to obtain

(A4) $$\begin{array}{l} \left\|h_{d}+\Phi v{\|}^{2}={\left(h_{d}+\Phi v\right)}^{H}(h_{d}+\Phi v\right)\\ \;\;\;\;\;\;\;\;\;\;\;\;\;\;\;\;\;\;\;\;={h_{d}}^{H}h_{d}+{h_{d}}^{H}(\Phi v)+{\left(\Phi v\right)}^{H}h_{d}+{\left(\Phi v\right)}^{H}(\Phi v) \end{array}$$

(1) First item ${h_{d}}^{H}h_{d}=\|h_{d}{\|}^{2}$; (2) The second and third items: ${h_{d}}^{H}(\Phi v)+{\left(\Phi v\right)}^{H}h_{d}$. These two terms are conjugate to each other (because the entire expression is real), so their sum is 2Re{vHΦHhd} or 2Re{hdHΦv} (the two are equivalent). By definition, $b=\Phi^{H}h_{d}$, so $v^{H}\Phi^{H}h_{d}=v^{H}b$, and thus the sum is 2Re{vHb}; (3) Fourth item: Since $A=\Phi^{H}\Phi$, ${\left(\Phi v\right)}^{H}(\Phi v)=v^{H}\Phi^{H}\Phi v=v^{H}Av$. This proves that

(A5) ‖hd+HbrΘhrv‖2=vHAv+2Re{vHb}+hd2

□
